# COVID-19 Operational Response Cell for the III Marine Expeditionary Force: An Interview With Navy Nurse LT Dana Brackup, NC, USN

**DOI:** 10.1093/milmed/usab224

**Published:** 2021-09-01

**Authors:** Dana M Brackup, Heather C King

**Affiliations:** 3rd Division Medical Battalion, Third Division Marine Logistics Group, Okinawa, Japan; TriService Nursing Research Program, Uniformed Services University, Bethesda, MD 20814, USA

## Abstract

During the coronavirus disease of 2019 (COVID-19) pandemic, overseas military bases faced unique challenges to preserve force health protection while simultaneously caring for military beneficiaries. The response to the rapidly evolving challenges surrounding transmission of the severe acute respiratory syndrome coronavirus 2 in Okinawa, Japan, required innovative solutions. One innovative solution was the COVID-19 Operational Response Cell established at Camp Courtney, Marine Corps Base Camp Smedley D. Butler. This interview describes the COVID-19 Response Cell operations and essential lessons learned by a Navy Nurse Corps officer working with III Marine Expeditionary Force, a forward-deployed force in the U.S. Indo-Pacific Command.

## INTRODUCTION

The emergence of severe acute respiratory syndrome coronavirus 2 (SARS-CoV-2) in 2019, quickly evolved into a worldwide pandemic. On March 11, 2020, the World Health Organization (WHO) declared coronavirus disease of 2019 (COVID-19) a worldwide pandemic.^1^ As of April 2021, WHO reported 138,688,383 confirmed cases of COVID-19 and 2,978,935 deaths attributed to COVID-19 globally.^1^ An immediate concern of U.S. military leaders was the threat to force health protection, operational readiness, and mission accomplishment from potential transmission of SARS-CoV-2 to military service members and their beneficiaries. Although military treatment facilities across the DoD quickly implemented the best available emerging evidence to respond to COVID-19, military bases located in Okinawa, Japan, faced unique challenges in responding to the COVID-19 pandemic. These challenges included language barriers to facilitate contact-tracing mechanisms, monitoring personnel movement, monitoring health risks, monitoring well-being of III Marine Expeditionary Force (MEF) service members and dependents, and optimizing interoperability among military and civilian stakeholders (Camp Foster, Kadena Air Base, Camp Courtney, Okinawa Ministry of Health, Defense Health Agency, and CDC).

The first SARS-CoV-2 case on the island of Okinawa, Japan, was identified in February 2020.^2^ Immediate contingency plans were initiated throughout the island to ensure personnel accountability for the health of service members and their beneficiaries. Additionally, the establishment of an Operational Response Cell by III MEF leadership at Camp Courtney in February 2020 provided a real-time solution to address SARS-CoV-2 contact tracing, implementation of restriction of movement, monitoring health risk assessment, and creating standard operating procedures for III MEF personnel.

Camp Courtney is a U.S. Marine camp located on MCB Camp Smedley D. Butler in Uruma City, Okinawa Prefecture, Japan. The III MEF, 3rd Marine Division, is located at Camp Courtney and provides the USA with a forward-deployed force in the U.S. Indo-Pacific Command. Camp Courtney is one of eight Marine Corps camps on Okinawa that support combat readiness. The readiness mission is accomplished by providing training, mobilization, and deployment support. During the COVID-19 pandemic, III MEF was tasked with maintaining the accountability, safety, and deployment readiness of forces.

## INTERVIEW QUESTIONS FOR EMERGENCY ROOM NURSE LT DANA BRACKUP DURING COVID-19

***As a Navy nurse working in the COVID-19 Operational Response Cell for III MEF, will you provide us with a summary of your education and training leading up to this unique assignment? What best prepared you for handling this important role during a pandemic?***

My entire Navy career has prepared me for responding to this COVID-19 crisis. After graduating from York College of Pennsylvania with a Bachelor of Science degree in Nursing, I was commissioned as a Navy Nurse Corps officer through the Nurse Candidate Program in December 2014. My first tour of duty was at Naval Medical Center Portsmouth, first working on the high-risk obstetrics/gynecology and postpartum unit and later transferring to the emergency department and becoming a certified emergency nurse with further training in trauma, burns, sexual assault, and en route care. From 2018 to 2020, I was assigned to the III MEF Marines in Okinawa, Japan, as the emergency/trauma nurse. As an officer in charge of the Medical Skills Training Center, I was selected to work at the COVID-19 Operational Response Cell as a qualified fleet Marine force warfare officer, a key leadership position in the COVID-19 operation and a unique role for a Navy nurse.

### What was the COVID-19 Operational Response Cell responsible for and What was the Team Composition?

The COVID-19 Operational Response Cell tracked COVID-19 results, quarantine periods, and restriction of movement for 53,000 service members and their dependents, to include III MEF members stationed in Hawaii. The COVID-19 Operational Response Cell executive leadership team included the commanding officer, a Marine Corps field officer, and a senior enlisted leader. This team provided direction and guidance for daily operations to include daily video conferences with general officers to discuss new guidance for dissemination at each III MEF unit. The staffing of the COVID-19 Operational Response Cell included three off-site military physicians (III MEF surgeon, preventive medicine, and infectious disease) who provided expertise and consultation, as needed, one on-site emergency room nurse, and one on-site corpsman. Weekly phone conferences with the III MEF surgeon and members of the COVID-19 Operational Response Cell provided essential information exchange and allowed the team to discuss issues and/or challenges. Additionally, military personnel included one representative from each subordinate unit of III MEF (3rd Marine Division, 3rd Marine Logistics Group, 3rd Marine Expeditionary Brigade, 31st Marine Expeditionary Unit, and 1st Marine Aircraft Wing). Other personnel at the COVID-19 Operational Response Cell included representatives from each staff section to include administrative, intelligence, operations, logistics, and signal support. COVID-19 Operational Cells were also located at Naval Hospital Okinawa and Kadena Air Base. Security surrounding the camp was provided by III MEF.

### How would you describe your contributions working in the Operational Response Cell as a Navy Nurse during the COVID-19 pandemic? What situations proved most challenging?

My unique set of skills as an emergency department nurse were an asset to the COVID-19 Operational Response Cell. The ability to think and act quickly, the capacity to communicate directly, confidently, and accurately, in addition to a wide range of emergency medical knowledge, were all qualities that helped me to excel in the COVID-19 Operational Response Cell. My recommendations and expertise for guidance were well received by senior and general officers as well as the commanding officer. My experience working in the COVID-19 Operational Response Cell broadened my understanding of the larger infrastructure for force health protection of service members, as I was actively involved in advocating for them.

At the COVID-19 Operational Response Cell, a database was developed and implemented to maintain accountability for all III MEF active duty, dependents, and civilians waiting for their COVID-19 test results or those who were quarantined. Data in the database include the name of service member and/or family members, III MEF subordinate unit, date of birth, gender, DoD identification number, symptoms (to include start date), travel information (location, airport, dates, etc.), date of COVID-19 test, date of COVID-19 result, test result (positive or negative), quarantine start date, quarantine end date, quarantine address, number of people in household, and any additional comments pertaining to the service member’s situation. Service members received a follow-up telephone call for confirmation of COVID-19 test results. During the first several months of the pandemic, obtaining COVID-19 test results took three-to-seven days. At the beginning of the pandemic, the U.S. Naval Hospital Okinawa did not have capabilities to evaluate COVID-19 tests, so the tests were mailed to Mainland Japan or South Korea for testing. Since the COVID-19 pandemic was a public health crisis, the Health Insurance Portability and Account Ability Act (HIPAA) was waived for reporting test results. This allowed for subordinate units of III MEF to have increased accountability of the health status for each of their service members and their families (both CONUS and OCONUS locations).

A daily COVID-19 medical risk assessment was provided to the COVID-19 Operational Response Cell commanding officer, which contributed to critical information and was provided directly to III MEF commanders in determining the appropriate liberty restriction in Okinawa, Japan. Within the same database, a tracking system for non-military affiliated local positive cases in Okinawa was created. This database helped to assess trends for COVID-19 positive cases in the area, which helped commanders decide if the area should be restricted for III MEF members and their families. Data collected in the database for the Okinawan population COVID-19 cases included gender, age, prefecture, positive test confirmation date, symptoms, and their occupation. The Okinawan patient information was taken directly from a local government-affiliated website, which tracked each positive COVID-19 case.^2^ Additionally, a weekly report of current contact tracing was sent to the COVID-19 Operational Response Cell from the Okinawa Prefecture Government to gauge future positive cases based on employment, travel, etc., and proved to be a valuable resource for the daily health risk assessment. The potential risks to the Status of Forces Agreement (SOFA) personnel were evaluated daily, sent to the COVID-19 Operational Response Cell commanding officer, and then sent to the III MEF commanding officer. Although information was de-identified, contact-tracing reports listed where each person was employed in Okinawa.

In the daily health risk assessment, updates on travel guidance by the Okinawan governor for the local Okinawa population were provided. It was composed of each day’s research of the local COVID-19 situation from local resources and the potential risk (low risk, moderate risk, or high risk) of exposure and/or infection to SOFA members. These data resulted in changes to the current restrictions for III MEF service members and their families in Okinawa.

The most significant challenge for this task was the language barrier. All local references were in Japanese. The COVID-19 Operational Response Cell team used websites to translate local news sources in addition to United States Naval Hospital’s translation services for up to date local information. Additionally, a Japanese intensive care unit nurse from a local Japanese hospital assisted in translation of news sources and documents. Another challenge included manning the shortage of nursing personnel. As the only nurse at the Operational Response Cell, I comprehended the data collection process created; however, initially, I did not have a plan for times when I was not physically at the Operational Response Cell. After realizing that I would not be able to work nonstop, I trained two corpsmen in the data collection process.

Another challenge was accurate reporting. Each of the original three operational cells on Kadena Air Base, Camp Courtney, and Naval Hospital Okinawa were collecting and reporting numbers (eventually to the same people in the chain of command), some service members and family members were accounted for more than once. This was recognized when a quality data review was conducted on the U.S. Naval Hospital Okinawa’s tracking system when access was granted. Initially, each of the three COVID response teams (18th Air Wing, Joint COVID-19 Response Cell (JCRC), and COVID Operational Response Cell) were collecting data and working asynchronously to develop a contingency plan. This made standard operating procedures, communication, and the delivery of care extremely challenging for the healthcare team, since each of these medical units did not have communication with the COVID-19 Operational Response Cell or each other initially. An enhanced communication plan or the creation of one COVID-19 Response Cell for Okinawa would have improved efficiency and decreased some of the challenges experienced.

**FIGURE 1.**
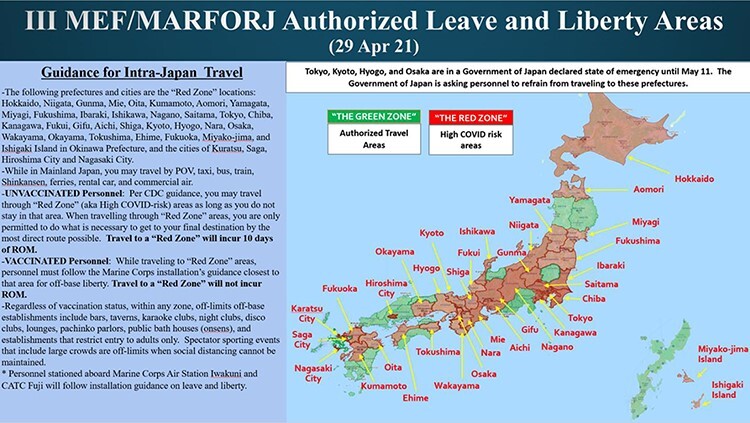
COVID-19 Operational Response Cell Trace and Clean Teams Process Active Duty, Civil Service, NAF, Contractor, Military Beneficiaries. April 2020.

From the service member perspective, it was challenging to receive up-to-date information in the rapidly evolving situation and with different standard operating procedures for bases located throughout the island. Each branch of service provided guidance on its unique individual restrictions. At one point in time, Navy personnel were allowed to go to spas or dine at a restaurant, while Marine Corps personnel were restricted from those activities. III MEF restrictions came directly from the COVID-19 Operational Response Cell, based on data from the daily health risk assessment and additional input from the commanding officer (Fig. 1).

**FIGURE 2.**
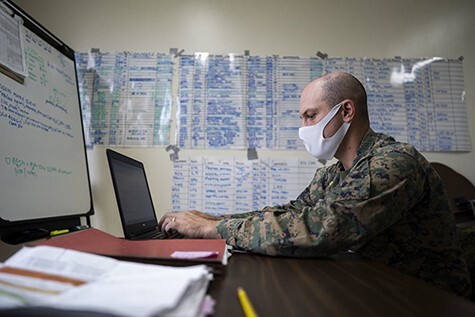
III MEF/MARFORJ Authorized Liberty Areas (indicated by green and red coloring) updated daily with data from COVID-19 Operational Response Cells. U.S. Marine Corps disseminated COVID-19 information, COVID-19 trace tracker, vaccination information, and links through an internet webpage https://www.iimef.marines.mil/coronavirus/.

**FIGURE 3.**
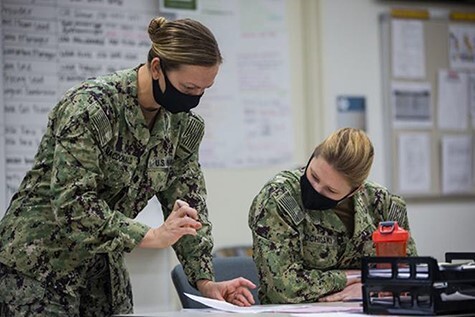
CDR Ann M. MacDonald, left, served as the data management lead with the Joint Operational COVID-19 Cell, and LCDR Stephanie P. Nochisaki, the Joint Operational COVID-19 Cell officer in-charge, review notes at the Public Health and Behavioral Health Center, Camp Foster, Okinawa, Japan. (U.S. Marine Corps photo by Cpl. Ryan H. Pulliam, VIRIN: 201,201 M-TX547-1097, Courtesy of the Media Defense.gov). Source: https://www.dvidshub.net/image/6442780/covid-one-year-later, Accessed 4.20.21.

**FIGURE 4.**
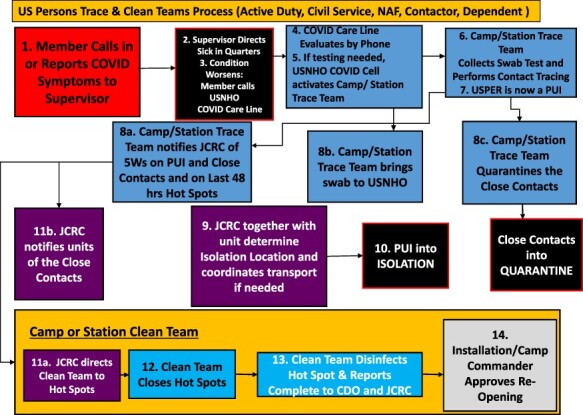
Maj Gregory Procaccini, the Joint COVID-19 Response Center Liaison Element officer in-charge with the COVID-19 Cell, drafts an email at the Public Health and Behavioral Health center, Camp Foster, Okinawa, Japan. (U.S. Marine Corps photo by Cpl. Ryan H. Pulliam, VIRIN: 201,202 M-TX547-1203, Courtesy of the Media Defense.gov). Source: https://www.dvidshub.net/image/6442784/covid-one-year-later, Accessed 4.20.21.

A final and important challenge was working collaboratively with multiple armed forces to achieve optimal interoperability for the COVID-19 response on Okinawa. The JCRC at U.S. Naval Hospital Okinawa was a great example of interoperability (Figs. 2 and 3). It was established at the start of the pandemic to provide Okinawa with a central hub for all COVID-19-related information for all forces located on the island (finding/stopping spread source). Initially, the joint aspect of the JCRC was the care and treatment of all SOFA members on Okinawa, not necessarily because of having joint service participation in the JCRC. Although the U.S. Naval Hospital took the lead for the majority of contact tracing throughout the island, the JCRC provided support to the Naval Hospital by finding key information and communicating findings to commanders.^3^ Concurrently, the COVID-19 Operational Response Cell had generated similar contact-tracing teams being executed with a mixture of medical and non-medical members from III MEF. The success of both contract tracing teams has allowed for the quarantine and isolation of COVID-19 positive and exposed individuals to decrease and slow the spread of the virus throughout Okinawa and successfully isolated two clusters of COVID-19 cases on Okinawa (Fig. 4). However, these teams could have further advanced collaborative efforts to collect and merge data, disseminate information, and streamline collective resources.

By June 2020, after I had relocated from the COVID-19 Operational Response Cell, one non-medical member from the team was selected to represent III MEF at the Joint Operational Response Cell at the Naval Hospital which laid the foundation for a joint response moving forward.

It was reported that two clusters of the virus were identified on Marine Corps Air Station Futenma and Camp Hansen. Contract tracing was credited for keeping COVID-19 positive cases strictly within the groups/clusters identified.

### Can you describe the communication between the Operational Response Cell and the JCRC in making this happen? Further explain the collaboration between military and Okinawa public health officials?

U.S. Naval Hospital Okinawa conducted case management, laboratory testing, epidemiology, and contact tracing, with the support from the JCRC. Contact tracing is the process of identifying people who may have come into close contact with an infected person.^3^ III MEF had established its own contact tracing teams used as a framework for incident management for Japanese or U.S. personnel who presented with symptoms of COVID-19 (Fig. 1). This was one of the first island-wide collaborative efforts between the COVID-19 Operational Response Cell and the JCRC at the Naval Hospital to combat COVID-19 in Okinawa. Each contact tracing team played an enormous role in preventing the COVID-19 positive cases within the group which was already identified.^3^ With the heavily enforced Marine Forces Japan policies and the close coordination with local health officials and the Naval Hospital, commands were able to successfully identify and isolate those who had been infected while keeping service members and their families away from the heavily populated COVID-19 positive areas throughout Okinawa.^3^

### Has the COVID-19 Pandemic Influenced Your Clinical Practices and How you Care for Patients?

The COVID-19 pandemic contributed to my development as a clinical nurse. When I have “down time,” I use this time to update anxious families in the waiting room who are not able to be at the bedside. I realize the importance of communicating with family members whenever possible. The “no visitor policy” for patients is still in place more than a year after the start of the pandemic. I also regularly conduct informal check-ins with members of my team who were quarantined (both from the COVID-19 Operational Response Cell and 3d Medical Battalion) with symptoms, traveling, etc. I make a point of checking in on fellow sailors—letting them know that I’m thinking about them and offering available resources.

### What Were the Most Valuable Lessons you Learned During COVID-19 That may Benefit Future Nurses Faced with Similar Issues in a Pandemic?

Specific to the COVID-19 Operational Response Cell

1. Interoperability with surrounding military forces (Navy, III MEF camps, Air Force, and Army) is a key in success and will reduce repetition of tasks and confusion. A synthesis of leadership and planning among each stakeholder and frequent communication among those on the island would enhance the response of SOFA members in Okinawa. Transparency regarding discovered resources between response cells would result in increased knowledge and readiness among forces.
2. Leveraging personnel with required language skills for translating information and communicating with key Okinawan stakeholders. Effective communication ensures a coordinated response for personnel located on military installations.
3. Developing a plan to check in with service members during prolonged periods of isolation and decreased interaction. In Okinawa, where many already feel isolated because of geographic location, this is important. III MEF commanders ordered all nonessential personnel to telework/isolate at home in an attempt to preserve the forces. Most off-base establishments were declared off-limits, to include restaurants, gyms, and salons. If employees in the on-base dining facilities became symptomatic and were tested for COVID-19, the services closed for cleaning. Boxed meals or Meals Ready to Eat were provided to marines and sailors in their barracks until proper sanitation of the facility had occurred. Service members had very little human interaction during this period, especially enlisted marines and sailors in the barracks. This combination of factors created greater isolation for service members. Proactively anticipating a plan for this may contribute to reducing mental health concerns during future pandemics.

## CONCLUSION

The formation of the COVID-19 Operational Response Cell by III MEF when presented with the threat of COVID-19 in Okinawa, Japan, in February 2020 greatly slowed the spread of infection among SOFA personnel. Challenges experienced initially with interoperability among III MEF, Naval Hospital Okinawa, and 18th Medical Group was a constant hurdle and affected how the preliminary pandemic response was handled among branches. Having an emergency department nurse working with a forward-deployed COVID-19 task force to tackle the pandemic response brought not only a unique perspective but also a valued medical expertise from non-medical III MEF leaders. Integrating and implementing the wealth of knowledge from Navy Nurse Corps officers will only strengthen the response and logistics to a future pandemic.
